# Supplementary material for: Sodium Nitrite Attenuates Reduced Activity of Vascular Matrix Metalloproteinase-2 and Vascular Hyper-Reactivity and Increased Systolic Blood Pressure Induced by the Placental Ischemia Model of Preeclampsia in Anesthetized Rats
Source: Int J Mol Sci. 2023 Aug 15;24(16):12818. doi: 10.3390/ijms241612818 (PMC10454117; doi:10.3390/ijms241612818)
Supplement: Supplementary file 1 [file ijms-24-12818-s001.zip › ijms-2505636-supplementary.pdf]

Table S1. Average of maternal, fetal, placental, vascular function and biochemical parameters.

Parameters	Group			
	**Norm-Preg**	**Preg+Nitrite**	**RUPP**	**RUPP+Nitrite**
Systolic blood pressure pregnancy day 21 (mmHg)	94 ± 2.4	78 ± 3.1 *	129 ± 6.9 *&	107 ± 5.4 #&
Fetal weight (g)	4.5 ± 0.1	3.6 ± 0.1 *	3.3 ±0.1 *&	4.2 ± 0.1 #&
Placental weight (g)	0.33 ± 0.007	0.32 ± 0.007	0.28 ± 0.006 *&	0.32 ± 0.011 #
Litter size (number of pups)	12.0 ±0.8	7.0 ± 2.0 *	7.0± 0.5 *	10.5 ± 1.0 #
Plasma levels of NO metabolites (mmol/L)	23 ± 2.8	37 ± 3.3 *	13 ± 0.3 *	15 ± 1.5 &
Placental levels of NO metabolites (μmol/100 mg of tissue)	158 ± 4.3	181 ± 8.5	218 ± 8.7 *&	229 ± 12.2 *&
Phe Emax (%) e+	1.6 ± 0.1	1.6 ± 0.1	2.9 ± 0.2 *&	2.2 ± 0.2 *#&
Phe Emax (%) e-	2.3 ± 0.3	2.4 ± 0.1	3.9 ± 0.3 *&	2.8 ± 0.2 #
Phe pEC_{50} (-log M) e+	6.5 ± 0.1	6.2 ± 0.1	7.1 ± 0.3	6.3 ± 0.3
Phe pEC_{50} (-log M) e-	7.0 ± 0.2	6.2 ± 0.1	8.3 ± 0.2	5.7 ± 1.1 #
ACh Emax (%) e+	87 ± 2.4	81 ± 3.7	52 ± 3.6 *&	56 ± 1.1 *&
ACh pEC_{50} (-log M) e+	6.0 ± 0.2	6.3 ± 0.4	4.2 ± 0.8	7.0 ± 1.3
72kDa MMP-2 (Arbitrary units)	0.32 ± 0.02	0.29 ± 0.03	0.09 ± 0.02 *&	0.20 ± 0.02 *#&
64kDa MMP-2 (Arbitrary units)	0.48 ± 0.04	0.44 ± 0.02	0.14 ± 0.01 *&	0.30 ± 0.03 *#&

Data are expressed as means ± SEM in Norm-Preg (n = 10), Preg+Nitrite (n = 10), RUPP (n = 10) and RUPP+Nitrite (n= 10) groups. *P < 0.05 vs Norm-Preg group; #P < 0.05 vs RUPP group and &P < 0.05 vs Preg+Nitrite group.

Zymography gel 1 of abdominal aorta

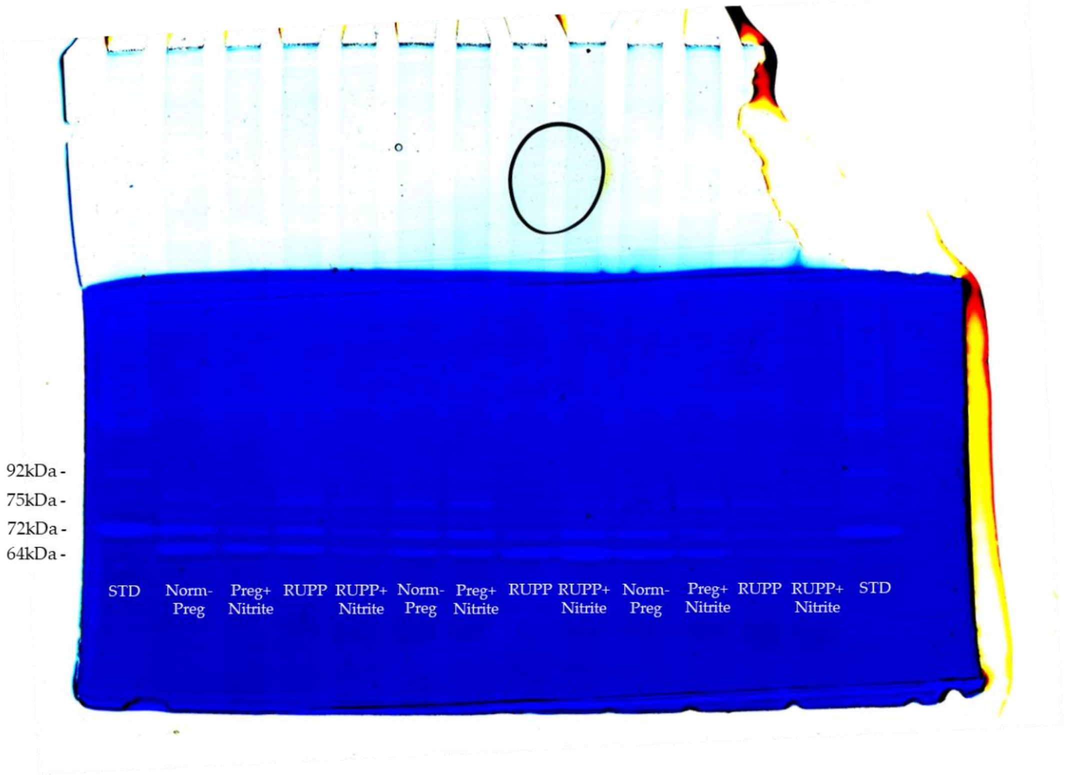

Supplementary Figure S1. Acrylamide gel stained with Coomassie Brilliant Blue to quantify the gelatinolytic activities of 92kDa MMP-9, 75kDa MMP-2, 72kDa MMP-2 and 64kDa MMP-2 in abdominal aorta samples of Norm-Preg, Preg+Nitrite, RUPP, and RUPP+Nitrite groups. STD: internal standard.

Zymography gel 2 of abdominal aorta

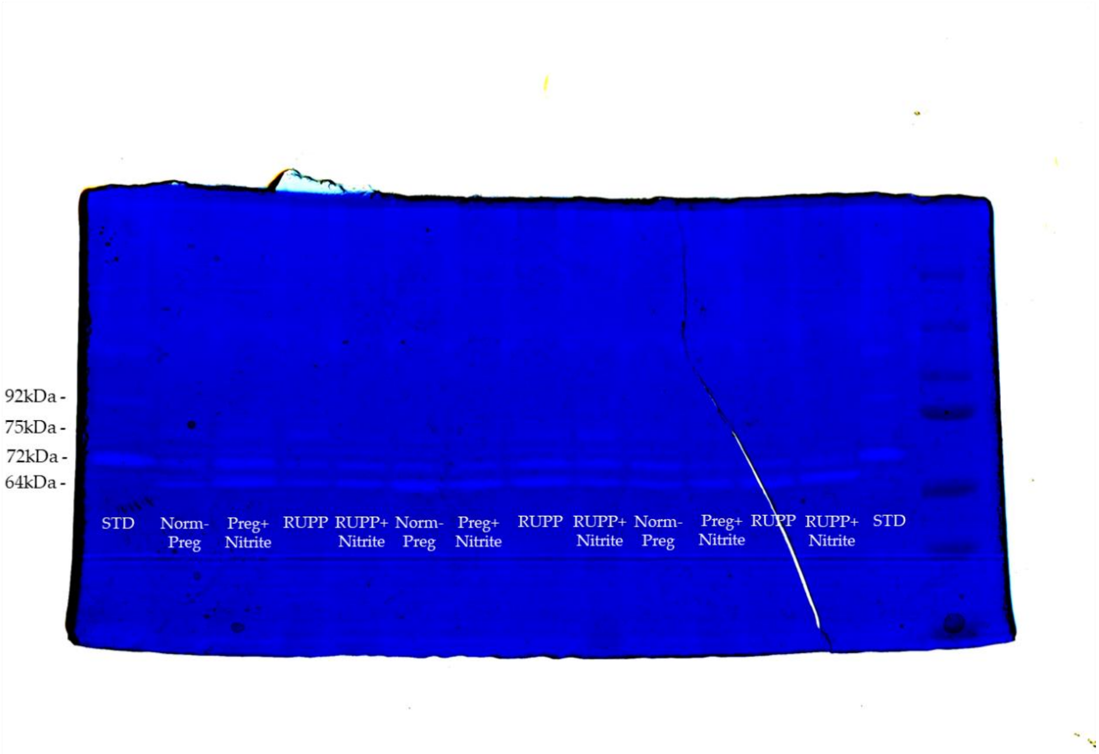

Supplementary Figure S2. Acrylamide gel stained with Coomassie Brilliant Blue to quantify the gelatinolytic activities of 92kDa MMP-9, 75kDa MMP-2, 72kDa MMP-2 and 64kDa MMP-2 in abdominal aorta samples of Norm-Preg, Preg+Nitrite , RUPP, and RUPP+Nitrite groups. STD: internal standard.

Zymography gel 3 of abdominal aorta

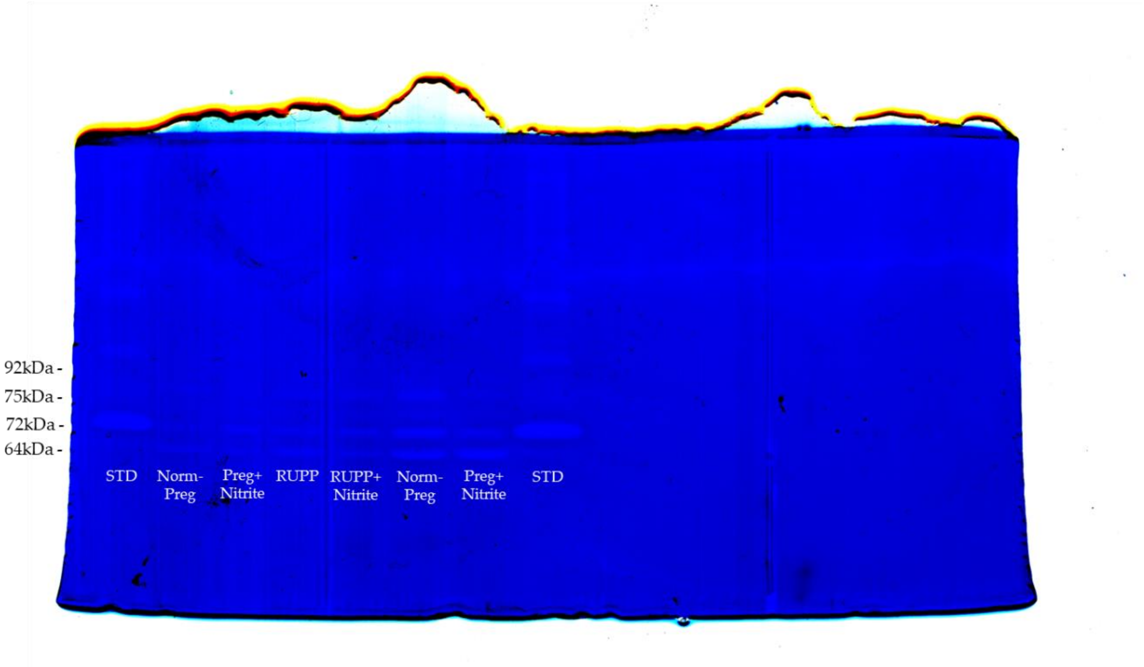

Supplementary Figure S3. Acrylamide gel stained with Coomassie Brilliant Blue to quantify the gelatinolytic activities of 92kDa MMP-9, 75kDa MMP-2, 72kDa MMP-2 and 64kDa MMP-2 in abdominal aorta samples of Norm-Preg, Preg+Nitrite , RUPP, and RUPP+Nitrite groups. STD: internal standard.
